# Cavitary Pneumonia: A Complication of Antibiotic Noncompliance

**DOI:** 10.1155/2020/5971348

**Published:** 2020-03-16

**Authors:** Jake L. Cotton, Tarig Ali-Dinar, Aledie Navas-Nazario

**Affiliations:** ^1^Department of Surgery, University of Central Florida College of Medicine, Orlando, FL, USA; ^2^Division of Pulmonology and Sleep Medicine, Nemours Children's Hospital, Orlando, FL, USA

## Abstract

In this report, we present a complicated case of community-acquired pneumonia in a 5-year-old boy. The patient first presented to the pulmonology clinic with the diagnosis of asthma and a recent history of recurrent pneumonia. Poor compliance to two courses of outpatient oral antibiotics resulted in persistent pneumonia symptoms with unresolved radiographic findings warranting parenteral antibiotics. Despite 2 symptom-free weeks, the patient returned to the emergency department with recurrence of symptoms where imaging revealed a cavitary lesion requiring a prolonged course of parenteral antibiotics. This report further supports the detrimental impact of partially treated infections related to poor compliance to antibiotic regimens.

## 1. Introduction

Patient noncompliance to systemic antibiotic treatment is known to have serious consequences and has been estimated to be between 39.4 and 50.5% in recent literature [1, 2]. Various factors play into antibiotic noncompliance including country, daily dosage regimen, patient age, physician attitude, and patient attitude to antibiotics, but it is often attributed to patient forgetfulness [1, 3]. Noncompliance is more likely among males and younger patients than their counterparts [1, 3]. It is well known that poor adherence may lead to complications including treatment failure, antibiotic resistance, additional hospital admissions, use of extra drugs, and deterioration of patients' health; however, there are few, if any, reports on the direct consequences on patient health from antibiotic noncompliance [3–5]. The World Alliance Against Antibiotic Resistance (WAAAR) has named education directed towards children/teenagers as one action to prevent the increasing prevalence of antibiotic resistance [6]. In this report, we present and discuss a case of simple community-acquired pneumonia that is often successfully treated as an outpatient, which was complicated with a chronic cavitary lesion requiring bronchoscopy and prolonged intravenous antibiotics with escalation of airway clearance and nebulized therapies. This result was deemed to be related to recurrent noncompliance to oral antibiotic treatment schedule. Risk factors such as immunodeficiency and other comorbidities have been investigated and ruled out through an extensive workup.

## 2. Case Presentation

A 5-year-old boy with a history of uncontrolled, moderate-persistent asthma presented to the pulmonology clinic for asthma management. The patient had a previously normal developmental history, up-to-date immunization record including Streptococcus pneumoniae and Haemophilus influenzae, and notable past medical history significant for recurrent otitis media due to poor compliance to oral antibiotics, which were subsequently successfully treated with parenteral antibiotics. Upon presentation, the patient recently had a left lower lobe community-acquired pneumonia that was treated with one dose of intramuscular ceftriaxone in the emergency department followed by a standard 10-day course of amoxicillin. The patient's family was instructed to open the capsule and mix the powder with food to improve compliance due to known history of poor palatability with previously prescribed antibiotics. The patient returned to the emergency department two weeks later with worsening of symptoms and was treated with oral cefdinir for unresolved pneumonia. He was subsequently seen in the pulmonology clinic four days later to establish care without acute complaint. He was started on fluticasone/salmeterol and a five-day course of oral prednisolone for management of an acute asthma exacerbation.

**Figure 1:**
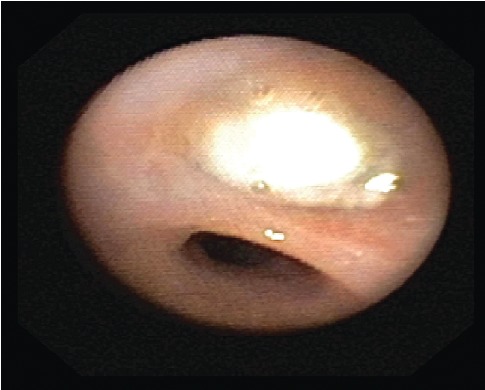


**Figure 2:**
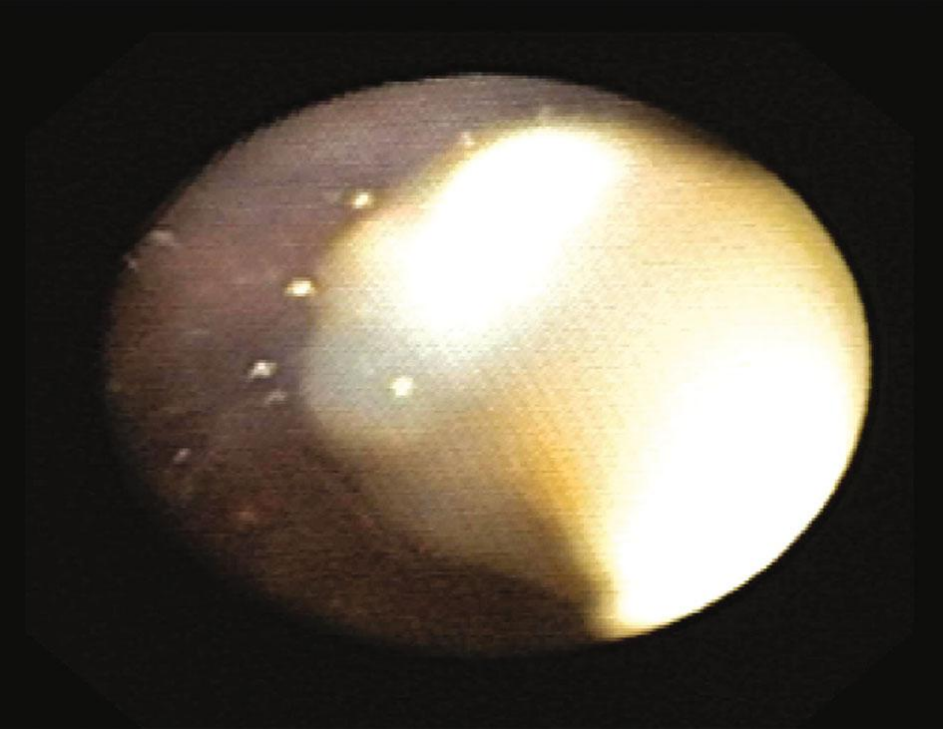


**Figure 3:**
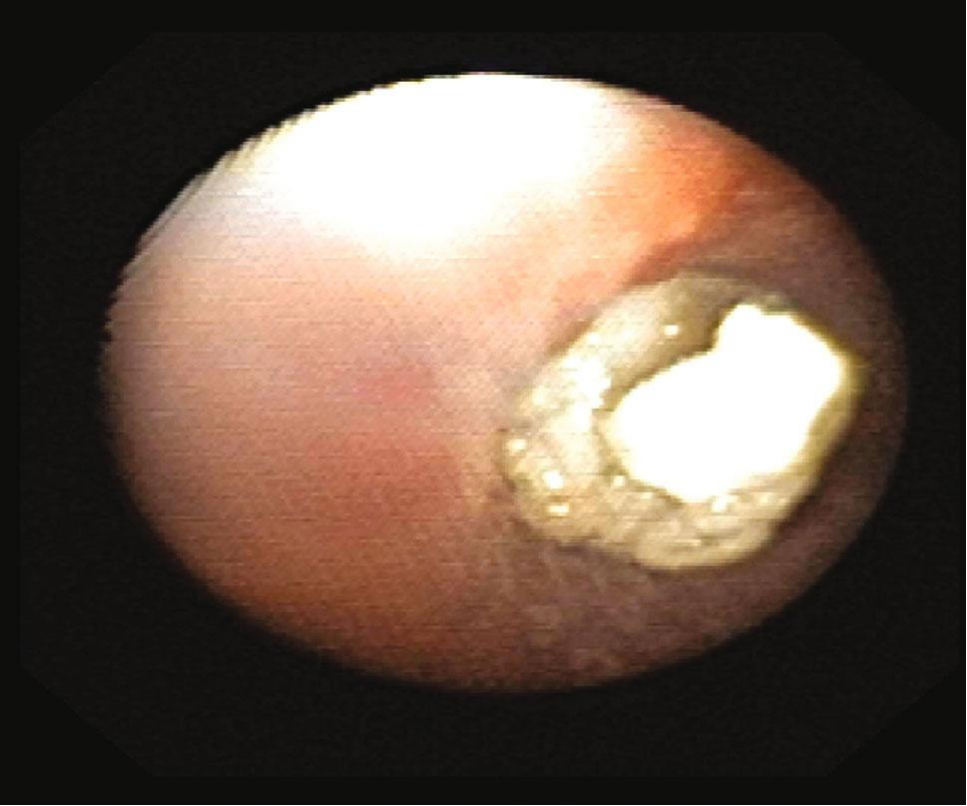


**Figure 4:**
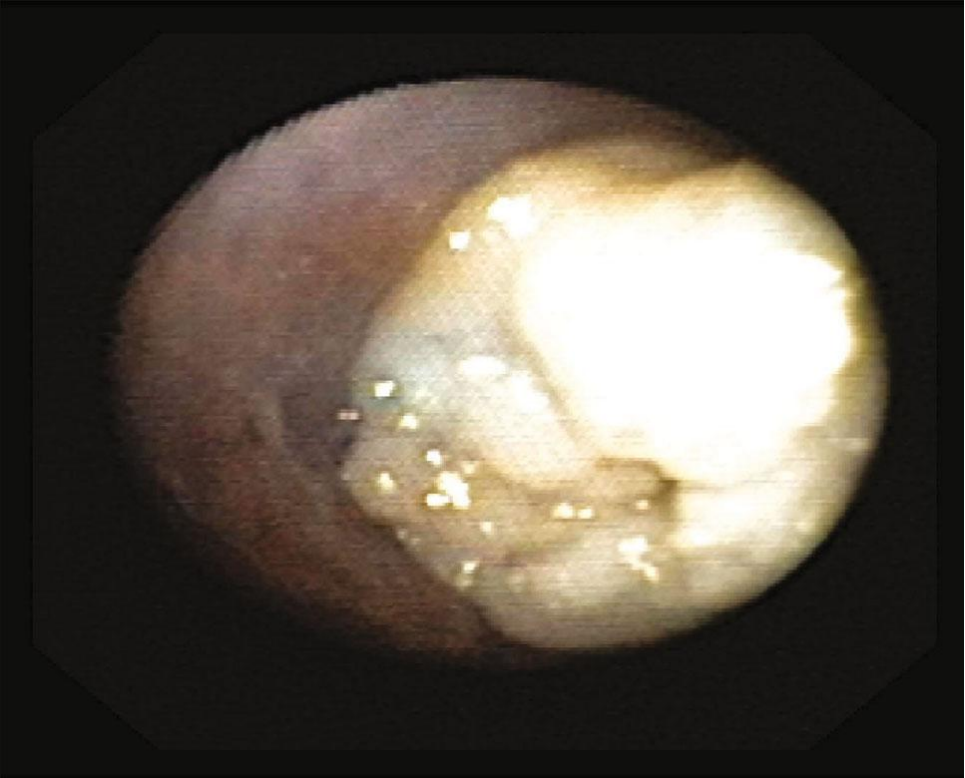


**Figure 5:**
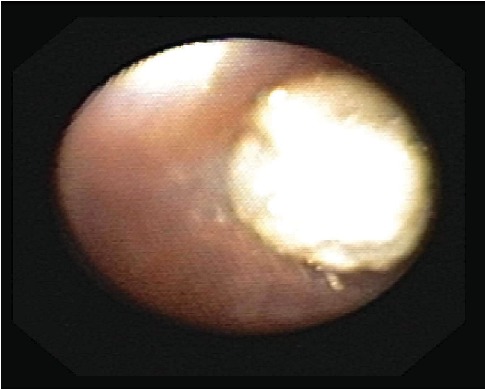


**Figure 6:**
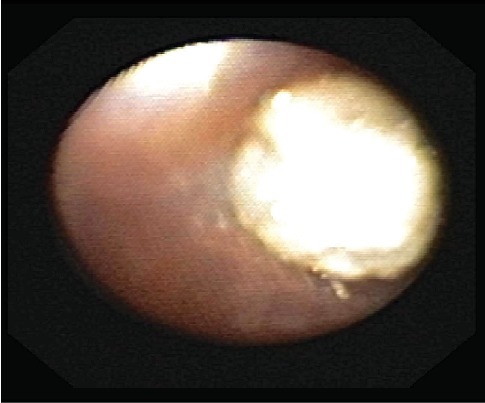


**Figure 7:**
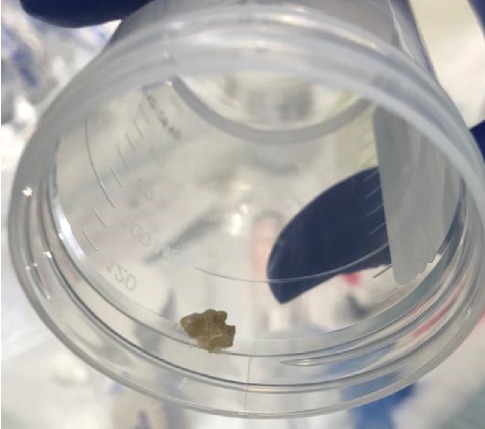


One month later, the patient presented to the emergency department with fever and increased cough and was diagnosed with right middle lobe pneumonia. He was treated with intramuscular ceftriaxone to be followed with a home course of oral antibiotics, which was reportedly completed. Two weeks later, the patient again presented to the emergency department with recurrent symptoms and diagnosed with right lower lobe pneumonia that failed outpatient treatment. The patient was subsequently admitted to the medical floor and managed with a single dose of intravenous ceftriaxone. He was discharged the following day in stable condition on cefdinir and azithromycin. The patient was to follow-up as an outpatient with his pulmonologist and primary care physician. Hospital laboratory work including CBC with differential, peripheral blood cultures, sweat chloride test, immunoglobulin assay, T cell count, and Aspergillus antibody was unremarkable. At the two-week follow-up, he had persistent fever, wheezing, daytime and nighttime cough, shortness of breath, exercise intolerance, and difficulty breathing. The patient was scheduled for further evaluation with high-resolution chest CT without contrast and flexible bronchoscopy with bronchoalveolar lavage and nasal cilia biopsy to evaluate for Primary Ciliary Dyskinesia (PCD). Repeat chest X-ray at that time revealed an improved right lower lobe consolidation without complete resolution, as expected. CT scan revealed a mild degree of bronchiectasis in the lower lobes, bilaterally, associated with consolidation on the right and linear atelectasis and scarring on the left with other nonspecific evidence of chronic inflammatory versus infectious process. Bronchoscopy identified multiple mucus plugs in the right bronchus intermedius (Figures 1–3), right middle lobe (Figures 4 and 5), and right lower lobe (Figure 6). Gross inspection of the bronchoscopy sample revealed a solid, rock-like mucus plug (Figure 7). Airway clearance via a high-frequency chest wall oscillation vest along with nebulized therapies was initiated. The patient did not tolerate postoperative oral azithromycin and required mixing with grape juice.

**Figure 8:**
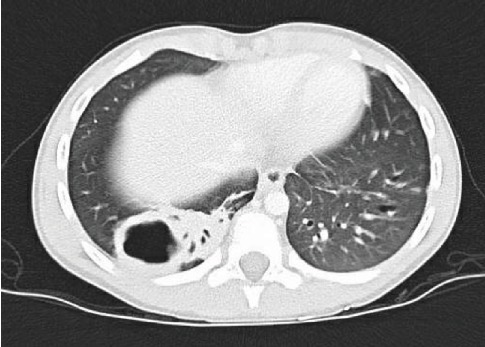


One week following the procedure, he was readmitted for recurrent fever and a new rounded lucency on chest X-ray in the right lung base, suspicious for cavitation or pneumatocele. Chest CT demonstrated a 2.5 × 3.2 × 3.7 cm area of cavitation within a region of consolidation or bronchiectasis in the posterior aspect of the right lower lobe with mediastinal and right hilar adenopathy, suspicious for cavitary bacterial pneumonia or fungal disease (Figure 8). Home treatment continued with the addition of intravenous ceftriaxone. Bronchoalveolar lavage culture from the previous week grew beta lactamase negative Haemophilus influenza and Moraxella catarrhalis. Fungal culture, mycobacterial culture, bronchial aspirate cell count, cytology, gastrin pepsin assay, and pathology report were all unremarkable. A PICC line was placed for 3 weeks of home antibiotics as a management for complicated cavitary pneumonia. Further workup included QuantiFERON-TB Gold, HIV assay, complement assay, tetanus antibody, diphtheria antibody, and pneumococcal antibody which were all unremarkable.

**Figure 9:**
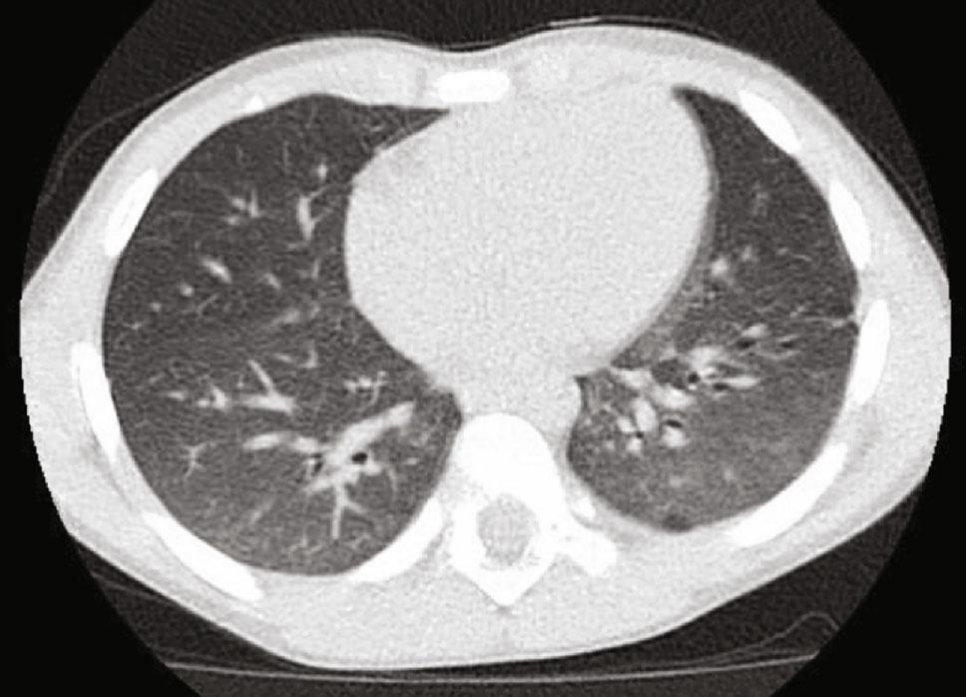


At 3-week follow-up post hospitalization, the patient was symptomatically improved without coughing or fever and he was reported to be back to his baseline. The mother reported adherence to antibiotic regimen through the PICC line. He continues maintenance therapy with fluticasone/salmeterol, montelukast, and vest therapy. He was started on oral azithromycin three times weekly. At the two-month follow-up, the patient remained asymptomatic, and repeat imaging at the nine-month follow-up revealed complete resolution of cavitating consolidation with residual bilateral mild bronchiectasis (Figure 9).

## 3. Discussion

In this case, we saw increased morbidity from a simple case of community-acquired pneumonia in an otherwise healthy 5-year-old patient. This resulted in more invasive treatment methods with significantly increased health care cost resulting from complications as bronchiectasis, despite treatment per the British Thoracic Society guidelines for the management of community-acquired pneumonia: update 2011. These recommendations include initial treatment with antibiotics when there is a clear clinical diagnosis of pneumonia, amoxicillin as first choice for oral antibiotic therapy, and macrolide supplementation if there is no response to first-line empirical therapy [7]. Despite these recommendations, one prospective study has found that most community-acquired pneumonia in children is viral in origin and bacterial superinfection is rare, only accounting for 15% of cases in children under the age of 18 [8]. To further compound this discordance, the addition of macrolides to beta-lactam therapy showed no statistically significant difference in length of hospital stay, intensive care admission, rehospitalization, or self-reported recovery [9]. These two studies support that select patients may see no benefit from beta-lactam monotherapy or dual therapy with the addition of a macrolide as in the presented case. Furthermore, the treatment of bacterial infections in pediatrics has been a controversial topic, and often, the route and duration of treatment recommended are determined by expert opinion and without appropriate clinical trials [10]. The final factor that may have led to increased morbidity in our patient is immunodeficiency. As discussed in the case presentation, our patient had no identifiable suggestion of immunodeficiency on laboratory testing; however, it is important to note that he had received a five-day course of prednisolone for management of asthma. Recently, there has been extensive work evaluating the complex and multifactorial genetics of the innate immune system that play a role in infection susceptibility, yet these are not routinely tested [11].

Compliance to oral antibiotic regimens can be challenging due to a multitude of reasons including patient misunderstanding and perceived improvement with symptom resolution prior to completion of an antibiotic course. This poses further barriers in the pediatric patient population due to patient preferences and poor education on the purpose of antibiotics. Beyond the issue of resistance, which has been well documented and studied, patient or physician noncompliance to an adequate treatment duration may result in an unfavorable outcome. In addition to the complication seen in this case, our patient was at increased risk for all side effects of imaging and treatment methods including high-resolution CT scan, bronchoscopy, central line placement, and prolonged intravenous antibiotics. We hope that our case will serve as a reminder and an example for physicians and patients alike of the consequences to erroneous and inadequate antibiotic treatment, regardless of the reason.

## References

[1] Vega-Cubillo E. M., Andres-Carreira J. M., Cirillo-Ibarguen S., Manzanares-Arnaiz C., Moreno-Moreno G., Redondo-Figuero C. G. (2017). Non-compliance with the systemic antibiotic treatment prescribed in primary health care emergency departments (study INCUMAT'). *SEMERGEN - Medicina de Familia*.

[2] Kardas P., Devine S., Golembesky A., Roberts C. (2005). A systematic review and meta-analysis of misuse of antibiotic therapies in the community. *International Journal of Antimicrobial Agents*.

[3] Pechere J. C., Hughes D., Kardas P., Cornaglia G. (2007). Non-compliance with antibiotic therapy for acute community infections: a global survey. *International Journal of Antimicrobial Agents*.

[4] Kardas P. (2002). Patient compliance with antibiotic treatment for respiratory tract infections. *The Journal of Antimicrobial Chemotherapy*.

[5] Bagnulo A., Munoz Sastre M. T., Kpanake L., Sorum P. C., Mullet E. (2019). Why patients want to take or refuse to take antibiotics: an inventory of motives. *BMC Public Health*.

[6] Carlet J. (2015). The world alliance against antibiotic resistance: consensus for a declaration. *Clinical Infectious Diseases*.

[7] Harris M., Clark J., Coote N. (2011). British Thoracic Society guidelines for the management of community acquired pneumonia in children: update 2011. *Thorax*.

[8] Jain S., Williams D. J., Arnold S. R. (2015). Community-acquired pneumonia requiring hospitalization among U.S. children. *The New England Journal of Medicine*.

[9] Williams D. J., Edwards K. M., Self W. H. (2017). Effectiveness of *β*-lactam monotherapy vs macrolide combination therapy for children hospitalized with pneumonia. *JAMA Pediatrics*.

[10] McMullan B. J., Andresen D., Blyth C. C. (2016). Antibiotic duration and timing of the switch from intravenous to oral route for bacterial infections in children: systematic review and guidelines. *The Lancet Infectious Diseases*.

[11] Netea M. G., van der Meer J. W. M. (2011). Immunodeficiency and genetic defects of pattern-recognition receptors. *The New England Journal of Medicine*.

